# Supervision Preparedness Among GP Registrars in the UK: A Survey Study

**DOI:** 10.12688/mep.21170.2

**Published:** 2026-03-19

**Authors:** Ioannis Saxionis, Bhargav Raut, Pritti Aggarwal, H. P. Patel

**Affiliations:** 1 University Hospital Southampton NHS Foundation Trust, Southampton, England, UK; 2 Living Well Practice, Southampton, UK; 3 University of Southampton, Southampton, England, UK; 4 NIHR Southampton Biomedical Research Centre, University of Southampton, Southampton, UK; 5 Academic Geriatric Medicine, University of Southampton, Southampton, UK

**Keywords:** clinical supervision; GP registrars; medical education; primary care training; supervisory preparedness

## Abstract

**Background:**

Clinical supervision (CS) plays a vital role in healthcare education and patient safety. As general practice embraces a multidisciplinary workforce, GP registrars are increasingly expected to supervise Allied Health Professionals (AHPs) and medical students from day one after qualifying. However, the extent of their preparedness for such roles remains unclear. Methods We conducted a cross-sectional online survey between August 2024 and January 2025 among UK GP registrars. The survey included quantitative and qualitative items assessing supervision experience, confidence, and perceptions of training. Descriptive statistics and content analysis were used to analyse the data.

**Results:**

A total of 52 registrars responded. The majority (n = 50 [96%]) reported no formal training on how to effectively supervise. Only 15% were supervising medical students, and n = 2 (4%) were supervising AHPs. Key barriers to effective supervision included lack of training, time constraints, and uncertainty about roles. Registrars expressed strong interest in structured training to build confidence in supervision.

**Conclusions:**

GP registrars report limited training and confidence in clinical supervision, despite growing expectations to supervise a diverse primary care team. Structured educational interventions as part of continuing professional development are needed to support supervision readiness and ensure safe, effective practice in a multidisciplinary environment.

## Introduction

Clinical supervision (CS) is a fundamental component of healthcare practice and education. One widely accepted definition describes it as the formal provision by a senior or qualified health practitioner, of an intensive, relationship-based educational interaction that is case-focused and designed to support, guide, and direct the clinical work of colleagues. ^1^ Across disciplines, CS is increasingly recognised not only as a means of safeguarding patient care but also of promoting reflective practice and professional development. ^2^ ^,^ ^3^ Effective CS has been linked to improved quality of care, ^4^ better staff performance, ^5^ and positive outcomes for both supervisees and the wider healthcare organisation. ^6^ ^,^ ^7^

The benefits of supervision are most pronounced when the process focuses on professional development and is facilitated by supervisors who possess the appropriate skills and clinical experience. ^8^ ^,^ ^9^ Supervisors who can build trusting relationships, offer meaningful feedback, and model competent practice tend to provide more impactful supervision. ^10^ However, finding supervisors with the appropriate expertise can be a significant challenge, particularly in busy clinical environments. ^11^ ^–^ ^13^ Time pressures, lack of protected teaching opportunities, and inconsistent supervisory training can undermine the quality and regularity of supervision offered. ^13^ ^–^ ^16^

These challenges are compounded in interprofessional contexts. In recent years, CS has increasingly taken place across professional boundaries in primary care, with GPs supervising not only foundation, specialty trainee doctors and medical students but also Allied Health Professionals (AHPs) which includes paramedics, advanced nurse practitioners, nurses and physician associates. ^17^ ^,^ ^18^ While this trend reflects the evolving nature of the primary care workforce, it brings unique complexities. The effectiveness of interprofessional supervision (IPS) is influenced by the clinical competence and confidence of both parties, and less experienced supervisees often report reduced feedback and difficulty asserting their discipline-specific needs. ^19^ Clear expectations, defined roles, and supportive infrastructure are essential for IPS to succeed. ^19^ ^,^ ^20^

GPs are expected to take on significant supervisory responsibilities, yet the training provided during GP specialty training often places limited emphasis on preparing registrars for these roles. Studies suggest that junior clinicians may lack a clear understanding of what clinical supervision entails ^5^ and that without appropriate training, supervision can become overly directive or even result in micromanagement, undermining autonomy and potentially threatening patient safety and quality of care. ^21^ At the same time, providing clinical supervision appears to be associated with lower burnout and increased job satisfaction. ^22^ Models such as restorative supervision have shown promise in supporting supervisee wellbeing, resilience, and motivation, ^23^ but their uptake within general practice remains limited.

Recognising these challenges, our study aims to explore the experiences, confidence levels, and perceptions of GP registrars regarding their readiness to supervise AHPs and medical students. The survey saw a higher response rate in Wessex owing to the authors’ training status there, which allowed stronger local promotion. By examining the extent of their training in supervision and identifying gaps, we hope to contribute to the ongoing dialogue on enhancing supervision standards and preparing future GPs for their essential roles as clinical supervisors.

## Methods

### Aim, design and setting

The aim of this study was to assess the experiences, confidence levels, and perceived preparedness of GP registars in the UK in supervising AHPs and medical students. This was a cross-sectional survey study, conducted from 11/8/24 to 3/1/25 via an online questionnaire. This study was conducted within the UK General Practice specialty training programme, a nationally standardised three-year postgraduate training pathway overseen by the Royal College of General Practitioners (RCGP).

### Participants

Participants included GP registrars at all stages of training, currently enrolled in a UK General Practice training programme. A total of 52 registrars completed the survey. Demographic information collected included stage of training, gender, ethnicity, and country of primary medical qualification. As dissemination occurred through multiple overlapping channels, the total number of registrars who were exposed to the survey invitation could not be accurately determined.

### Ethics and consent

This study was conducted as an educational evaluation of GP training experiences and did not involve patients, patient data, or any intervention. In line with UK Health Research Authority (HRA) guidance, formal NHS Research Ethics Committee approval was not required, as the survey collected anonymised data from healthcare professionals about their educational experiences and perceptions. Participation was voluntary, and informed consent was implied through completion and submission of the questionnaire. No participant identifiable data were collected.

### Survey tool development and data collection

An online survey comprising 20 questions including multiple-choice and Likert-style questions, alongside short free-text questions to allow respondents to elaborate on perceived barriers and suggestions for improvement was developed using Microsoft Forms. The survey aimed to explore GP registrars’ experiences with supervision, their perceived confidence in supervisory roles for both AHPs and medical students, and their perspectives on supervision training. The survey included a combination of multiple-choice questions assessing formal training received, current supervisory responsibilities, and frequency of supervision, as well as open-text questions exploring perceived challenges and suggestions for improving supervision training.

The survey link was disseminated to GP registrars through a multi-faceted approach, including posts on closed social media platforms used by GPregistrars, and during scheduled GP teaching sessions. Participation was voluntary. Anonymity was maintained throughout the study, with no personally identifiable information directly linked to the survey responses.

The survey instrument was developed by the authors following a review of existing literature on clinical supervision, interprofessional supervision, and supervision training within healthcare education. It measured four constructs: preparedness (“How prepared do you feel to supervise trainees?”, 0–10 scale), confidence (“How confident are you in providing effective supervision?”, 0–10 scale), barriers (free-text responses), and supervisory behaviours (5-point never-always scale for teaching/feedback frequency). No pilot testing was conducted. Prior to dissemination, the survey was reviewed by senior academic GPs with expertise in medical education to assess face validity and content relevance. As this was an exploratory educational survey, formal validation was not undertaken.

### Data analysis

Quantitative data were analysed descriptively using Microsoft Excel. Responses were reported as counts and percentages. Open-text responses were analysed thematically to identify common experiences and suggestions.

### Data availability

The updated dataset is available on Figshare: *Saxionis, Ioannis; Raut, Bhargav (2025). Survey regarding supervision training as a GP registrar.xlsx.* Figshare. Dataset. https://doi.org/10.6084/m9.figshare.29366741.v3.

The extended data include the survey questionnaire and are available on the same Figshare record.

## Results

Following distribution, a total of 52 GP registrars completed the online survey.

### Supervision training and experience

Most respondents (n = 50, 96%) reported not having received any formal training or guidance on how to supervise others in their GP practice. Only 2 registrars (4%) indicated they had received such training. At the time of the survey, 8 registrars (15%) were currently supervising medical students, and 2 registrars (4%) were supervising allied health professionals. The frequency of supervision varied, with the largest proportion of respondents (n = 31, 60%) not currently supervising anyone. Sixteen registrars (31%) reported supervising rarely, 3 (6%) supervised monthly, and 1 respondent each (2%) reported weekly and daily supervision.

### Content analysis

Free-text responses describing experiences and perceptions of supervision were analysed descriptively. Four key themes were identified.

• Time constraints

The most reported barrier to supervision was lack of time, with many registrars describing competing clinical demands that limited opportunities for teaching or feedback. As one respondent noted, *“There are no dedicated supervision slots in the schedule,”* while another highlighted that supervision felt difficult *“alongside full clinical workload.”*

• Lack of training

Thirty-nine respondents described an absence of formal preparation for supervisory roles. Several explicitly stated that they had received no training at all, for example: *“I have no supervision training or opportunity to do so,”* and *“No training so far so no idea.”* Others suggested that supervision should be formally introduced during GP training, such as *“Inclusion of supervision in VTS sessions.”*

• Confidence

Nineteen registrars expressed a lack of confidence in supervising others, often linked to limited experience and training. One respondent commented, *“It is difficult to know what should be expected of different roles,”* while another felt that confidence would improve with *“supported supervisor sessions in final months of training.”*

• Challenges related to supervisees

Thirteen respondents identified challenges related to supervisees, including variable knowledge levels and uncertainty around scope of practice, particularly for AHP. One registrar noted, *“It is difficult to know what types of things should be expected of a PA,”* highlighting uncertainty around role boundaries.

A small number of participants were unsure about barriers to supervision (n = 4) or reported other factors (n = 3).

### Training background

Over half of the respondents (n = 28, 54%) had between 0 and 5 years of postgraduate training experience. Eighteen registrars (35%) reported having 5 to 10 years of experience, and 6 registrars (11%) had more than 10 years of postgraduate training. The primary medical qualifications of the respondents were diverse, with 20 (38%) obtained from the United Kingdom. Other countries represented included India (n = 9, 17%), Pakistan (n = 3, 6%), Nepal (n = 2, 4%), Egypt (n = 2, 4%), and single respondents (2% each) from Iraq, Ghana, Nigeria, South Africa, Italy, Turkey, and Portugal.

### Demographic characteristics

The demographic characteristics of the survey respondents are summarized in Table 1.

**Table 1.** Demographic Characteristics of Survey Respondents (N = 52).

Characteristic	Category	n (%)
**Gender**	Female	27 (52%)
	Male	23 (44%)
	Non-binary	1 (2%)
	Preferred not to say	1 (2%)
**Ethnicity**	White	23 (44%)
	Asian or Asian British	15 (29%)
	Black or Black British	2 (4%)
	Mixed/Multiple ethnic groups	3 (6%)
	Other ethnic group	5 (10%)
	Preferred not to say	4 (8%)
**Gender & Ethnicity Combination**	Female – White	15 (29%)
	Male – Asian or Asian British	8 (15%)
	Male – White	8 (15%)
	Female – Asian or Asian British	7 (13%)
**Training Stage**	ST1	6 (12%)
	ST2	17 (33%)
	ST3	27 (52%)
	Alternative	2 (4%)
**Deanery**	Wessex	25 (48%)
	East of England	8 (15%)
	West Midlands	5 (10%)
	Peninsula	4 (8%)
	Other UK Regions	10 (19%)

### Training stage and deanery

Over half of the respondents were in their final year of GP training (ST3, n = 27, 52%). Seventeen registrars (33%) were in ST2, and 6 (12%) were in ST1. Two respondents (4%) identified as being in an alternative training stage. Most respondents were based in the Wessex deanery (n = 25, 48%). Other participating deaneries included the East of England (n = 8, 15%), West Midlands (n = 5, 10%), Peninsula (n = 4, 8%), and other UK regions (n = 10, 19%).

## Discussion

This study provides a valuable insight into the supervision preparedness of GP registrars in the UK, highlighting a significant lack of formal training in this crucial aspect of their future roles. The finding that only a small minority of registrars had received any specific guidance on supervision underscores a potential gap in current GP training programs. This is particularly pertinent given the increasing integration of AHPs into primary care teams, requiring GPs to effectively supervise a diverse range of colleagues. ^17^ ^,^ ^18^

Our findings align with existing literature that emphasises the importance of clinical supervision for maintaining patient safety and promoting reflective practice, ^2^ while also acknowledging the challenges posed by lack of protected clinical teaching time constraint on busy clinical staff. ^16^ The open-text responses from registrars echoed these concerns, with “lack of training” and “time constraints” being the most frequently cited perceived challenges in supervising others, which mirrors findings from other studies, where lack of time has been consistently identified as a key barrier to effective supervision. ^13^ This reinforces the need to address these barriers to ensure effective supervision. The benefits of supervision, as perceived by the registrars, included holistic professional development, opportunities for learning and knowledge consolidation, and contributing to the education of future clinicians. These positive perceptions suggest a willingness among registrars to engage in supervisory roles, provided they feel adequately prepared and supported. However, the limited awareness of available resources to support supervision further emphasises the current lack of formal integration of supervision training within their curriculum.

The need for better recognition of clinical supervision within organisational policy, as highlighted by AHPs, ^9^ is also relevant to the GP registrar experience. Inadequate preparation for supervision can lead to a lack of confidence, potentially fostering micromanagement, ^21^ which is not only inefficient but can also undermine trust.

The open-text responses also revealed a desire for increased training in supervision, ideally integrated into the existing GP training scheme. Suggestions included dedicated supervision sessions, learning through teaching opportunities (e.g., supervising medical students or more junior doctors under guidance), and clear guidance on available resources and legal responsibilities.

### Limitations

This study is one of the first to examine GP registrars’ experiences of supervision during training, capturing perspectives from both quantitative and qualitative data. The mixed-methods design allowed us to identify common patterns while also exploring trainees’ explanations in their own words. The findings have direct relevance to training programmes and workforce development.

However, several limitations should be considered. The cross-sectional design captures perceptions at a single point in time, and the modest sample size (n = 52) may limit generalisability to the wider UK registrar population. While the survey was disseminated widely through GP teaching sessions, WhatsApp groups, and social platforms, the total number of registrars reached is unknown and therefore a formal response rate could not be calculated.

Most respondents were from the Wessex deanery, which may introduce regional bias; however, given the standardisation of the GP training curriculum across the UK, it is possible that the findings may be cautiously extrapolated to registrars nationally.

In addition, qualitative data were derived from short free-text responses, which limited the depth of content analysis and precluded follow-up to clarify or expand on emerging themes. Voluntary participation and snowball distribution may have introduced selection bias, whereby registrars with stronger views or experiences of supervision may have been more likely to respond, in line with recognised limitations of online surveys as described in CHERRIES guidance. ^24^

## Conclusions

This study demonstrates a clear need to consider the formal integration of education and training in clinical supervision for GP registrars. The significant lack of reported supervision training amongst GP registrars highlights a crucial gap that needs to be addressed to prepare future GPs for their evolving supervisory responsibilities, particularly concerning AHPs. Addressing this gap through structured training programs, as suggested by the registrars themselves, has the potential to benefit supervisees through more effective guidance and support, supervisors by enhancing their confidence and skills, and ultimately patients through more efficient and safer care delivery. The integration of supervision training within the GP curriculum, along with providing accessible resources and protected time for supervision, is important to foster a positive and effective learning environment within primary care. Future research should explore the impact of specific supervision training interventions on both the confidence and competence of GP supervisors and the experiences of their supervisees.

## Declarations

HPP has received lecture fees from Abbott, Pfizer, and HC-UK conferences outside of the submitted work. PA is the Director for Primary Care Education for Medical Students and is also a GP trainer. IS and BR declare no competing interests.

Generative AI (ChatGPT 5.4) was used solely for grammatical corrections, phrasing improvements, and language polishing of author-written content throughout the manuscript. All conceptual content, data analysis, interpretations, and scientific arguments remain the original work of the authors.

## Author contributions

IS, BR and PA participated in the conception and conduct of the study. IS and BR coordinated the survey, conducted the data analysis, and drafted the initial version of the manuscript. All authors reviewed and edited the final version of the manuscript.
