# Supplementary material for: Supervision Preparedness Among GP Registrars in the UK: A Survey Study
Source: MedEdPublish (2016). 2026 Mar 19;15:194. Originally published 2025 Oct 8. [Version 2] doi: 10.12688/mep.21170.2 (PMC13019035; doi:10.12688/mep.21170.2)
Supplement: Peer review history

# Reviewer report: Reviewer response for version 2

**DOI:** 10.21956/mep.23080.r45597

**Published:** 2026-05-12

**Authors:** Devi Prasad Mohapatra

**Affiliations:** 1 Jawaharlal Institute of Postgraduate Medical Education & Research (JIPMER), Puducherry, Puducherry, India

I approve the manuscript.

Have any limitations of the research been acknowledged?

Yes

Is the work clearly and accurately presented and does it cite the current literature?

Yes

If applicable, is the statistical analysis and its interpretation appropriate?

Yes

Are all the source data underlying the results available to ensure full reproducibility?

Yes

Is the study design appropriate and is the work technically sound?

Yes

Are the conclusions drawn adequately supported by the results?

Yes

Are sufficient details of methods and analysis provided to allow replication by others?

Partly

Reviewer Expertise:

Curriculum Development, continuing professional development, digital tools in medical training, simulation

I confirm that I have read this submission and believe that I have an appropriate level of expertise to confirm that it is of an acceptable scientific standard.

# Reviewer report: Reviewer response for version 2

**DOI:** 10.21956/mep.23080.r45598

**Published:** 2026-03-25

**Authors:** Prof. Irma Manjavidze M.D.,PhD., Associate Professor. Dali Chitaishvili M.D., PhD.

**Affiliations:** 1 Head of Department of Clinical Skills and Multidisciplinary simulation, Tbilisi State Medical University, Tbilisi, Georgia; 2 Clinical Skills and Multidisciplinary Simulation Department, Tbilisi State Medical University, Tbilisi, Georgia

We have reviewed the revised manuscript and the authors’ detailed responses, and we are satisfied that our previous concerns have been adequately addressed. We would like to update our decision to **Approved**

Have any limitations of the research been acknowledged?

Yes

Is the work clearly and accurately presented and does it cite the current literature?

Yes

If applicable, is the statistical analysis and its interpretation appropriate?

Yes

Are all the source data underlying the results available to ensure full reproducibility?

Yes

Is the study design appropriate and is the work technically sound?

Yes

Are the conclusions drawn adequately supported by the results?

Yes

Are sufficient details of methods and analysis provided to allow replication by others?

Partly

Reviewer Expertise:

Simulation based learning, Medical Education, Clinical Skills, pediatrics

We confirm that we have read this submission and believe that we have an appropriate level of expertise to confirm that it is of an acceptable scientific standard.

# Reviewer report: Reviewer response for version 1

**DOI:** 10.21956/mep.22676.r44947

**Published:** 2026-01-10

**Authors:** Prof. Irma Manjavidze M.D.,PhD., Associate Professor. Dali Chitaishvili M.D., PhD.

**Affiliations:** 1 Head of Department of Clinical Skills and Multidisciplinary simulation, Tbilisi State Medical University, Tbilisi, Georgia; 2 Clinical Skills and Multidisciplinary Simulation Department, Tbilisi State Medical University, Tbilisi, Georgia

Overall assessment

 This manuscript addresses a timely and operationally important issue: the preparedness of GP trainees/registrars to undertake supervisory roles (medical students and an increasingly multidisciplinary primary care workforce). The rationale is well developed, and the study design (cross-sectional online survey with free-text items) is broadly appropriate for exploring perceived preparedness, confidence, and training exposure. The headline finding—very limited formal supervision training reported by respondents—has clear implications for curriculum design and workforce governance.

 That said, several aspects require strengthening to support interpretability, reproducibility, and appropriate generalisation: (1) survey development/validity and reporting aligned to survey standards; (2) sampling frame/denominator and recruitment transparency; (3) substantially more detail on the qualitative analytic approach; (4) clearer governance/ethics justification; and (5) some internal inconsistencies (notably declarations/competing interests and terminology).

 If these points are addressed, the paper could make a useful contribution as a needs assessment to inform structured supervision training in GP specialty training and continuing professional development.

 3) Are sufficient details of methods and analysis provided to allow replication by others?

**Partially.**

 Partial—quantitative methods are broadly replicable, but qualitative/thematic methods are under-described and need more detail to replicate.

**Major comments **

1. **Population and sampling**
  Clarify whether the survey was Wessex-focused or multi-deanery, describe recruitment and approximate reach, and discuss potential selection bias in line with CHERRIES.
2. **Survey validity**
  Briefly describe survey development, piloting, constructs measured, and how confidence/preparedness were assessed, with a simple item–construct mapping if possible.
3. **Ethics and governance**
  Expand the UK rationale for no ethics approval and clarify consent, data handling, GDPR compliance, storage, and confidentiality safeguards.
4. **Qualitative methods**
  Provide more detail on thematic analysis methods, coding processes, meaning of theme counts, and include anonymised exemplar quotes.
5. **Definition of AHPs**
  Clearly define which professional roles are included under “AHPs” or consider broader MDT terminology.
6. **Competing interests**
  Ensure consistency between the competing interests statement and the declarations section.
7. **Quantitative reporting**
  Report missing data and denominators per item and consider simple descriptive subgroup summaries.

**Minor comments and editorial suggestions.**

 Use unambiguous date formats and consistent terminology for the participant group, and temper outcome language so conclusions reflect perceptions/training exposure rather than demonstrated safety outcomes. Consider simplifying or moving potentially identifying demographic cross-tabulations to supplementary material and explicitly referencing CHERRIES to strengthen reporting transparency.

**Recommendation: Major revisions.**

 The topic is important and the findings are potentially indexable; however, the manuscript requires clearer governance/ethics justification, stronger survey reporting (including sampling/denominators), and substantially more detail and supporting evidence for the qualitative analysis. Addressing these points will improve methodological credibility, reproducibility, and confidence in the conclusions.

Have any limitations of the research been acknowledged?

Yes

Is the work clearly and accurately presented and does it cite the current literature?

Yes

If applicable, is the statistical analysis and its interpretation appropriate?

Yes

Are all the source data underlying the results available to ensure full reproducibility?

Yes

Is the study design appropriate and is the work technically sound?

Yes

Are the conclusions drawn adequately supported by the results?

Yes

Are sufficient details of methods and analysis provided to allow replication by others?

Partly

Reviewer Expertise:

Simulation based learning, Medical Education, Clinical Skills, pediatrics

We confirm that we have read this submission and believe that we have an appropriate level of expertise to confirm that it is of an acceptable scientific standard, however we have significant reservations, as outlined above.

# Response

**Published:** 2026-03-09

**Authors:** Ioannis Saxionis

**Affiliations:** Primary Care, University Hospital Southampton NHS Foundation Trust, Southampton, England, UK

We thank the reviewer for their thoughtful and detailed feedback. We appreciate the recognition of the relevance of this topic and the potential contribution of the study as a needs assessment for improving supervision training in GP specialty training. We have revised the manuscript to address the reviewer’s comments and improve clarity, transparency, and reproducibility. Our responses are provided below:

 1. Population and sampling Comment: 

 Clarify whether the survey was Wessex-focused or multi-deanery, describe recruitment and approximate reach, and discuss potential selection bias in line with CHERRIES.

 Response: 

 The Methods section has been expanded to clarify the recruitment strategy and survey dissemination process. The survey was distributed through GP training networks and professional communication channels, which included registrars from multiple regions; however, the highest response rate was observed in the Wessex region, reflecting the authors’ professional networks rather than an intentional sampling focus. This has now been clarified in the manuscript. Because the survey was disseminated through multiple overlapping channels, it was not possible to determine a precise denominator or calculate a formal response rate. This limitation and the potential for selection bias have now been explicitly acknowledged in the Methods and Limitations sections, in line with good practice for reporting online surveys.

 2. Survey validity Comment: 

 Describe survey development, piloting, constructs measured, and how confidence/preparedness were assessed.

 Response: 

 The survey development process is now described in greater detail in the Methods section. The questionnaire was developed following a review of existing literature on clinical supervision. The survey included items assessing four main constructs: preparedness, confidence, perceived barriers, and supervisory behaviours. Preparedness and confidence were assessed using 0–10 rating scales, while supervisory behaviours were assessed using Likert-type scales. The questionnaire was reviewed by senior academic GPs with expertise in medical education to assess content relevance prior to dissemination. As this study aimed to provide an exploratory needs assessment, formal psychometric validation and pilot testing were not undertaken, which is now acknowledged as a limitation.

 3. Ethics and governance Comment: 

 Clarify the rationale for no formal ethics approval and expand information regarding consent, data handling, GDPR compliance, and confidentiality safeguards.

 Response: 

 The ethical and governance considerations have been expanded in the Methods section. In accordance with UK Health Research Authority (HRA) guidance, this study was classified as service evaluation/educational research involving healthcare professionals rather than patients, and therefore formal NHS Research Ethics Committee approval was not required. Participation in the survey was voluntary and anonymous, and completion of the survey constituted informed consent. No identifiable personal data were collected. Data were stored securely and handled in accordance with institutional data protection policies and UK GDPR requirements. These details have now been clearly stated in the revised manuscript.

 4. Qualitative methods Comment: 

 Provide more detail on thematic analysis methods, coding processes, and include quotes.

 Response: 

 We agree that additional methodological clarity was needed. The manuscript has been revised to clarify that the qualitative analysis used content analysis rather than thematic analysis, and the description of the analytic approach has been expanded. Free-text responses were reviewed to identify recurring concepts and patterns, which were grouped into broader categories representing key themes in the responses. In addition, representative anonymised quotes from respondents have now been included in the Results section to illustrate these themes and improve transparency of interpretation.

 5. Definition of AHPs Comment: 

 Clarify which roles are included under Allied Health Professionals (AHPs). Response: 

 The Introduction has been revised to clarify the multidisciplinary context of primary care and to specify examples of professionals included within the supervisory scope in general practice, such as paramedics, nurses, advanced practitioners and physician associates.

 6. Competing interests Comment: 

 Ensure consistency between the competing interest's statement and declarations.

 Response: 

 The competing interests and declarations sections have been reviewed and revised to ensure consistency and clarity across the manuscript.

 7. Quantitative reporting Comment: 

 Report missing data and denominators per item and consider subgroup summaries. Response: 

 The quantitative results section has been clarified to improve reporting transparency, including clearer presentation of response numbers for survey items. Given the relatively small sample size, the analysis remains descriptive, and subgroup analyses were not performed in order to avoid over-interpretation. This is acknowledged in the limitations.

 8. Minor editorial suggestions Comment: 

 Use consistent terminology, clearer date formats, temper outcome language, and consider referencing CHERRIES.

 Response: 

 The manuscript has been revised to ensure consistent terminology (“GP registrars” used throughout) and clearer date formatting.  Additionally, CHERRIES has been referenced as suggested We thank the reviewer again for their detailed and constructive feedback.

# Reviewer report: Reviewer response for version 1

**DOI:** 10.21956/mep.22676.r44209

**Published:** 2025-12-29

**Authors:** Ardi Findyartini

**Affiliations:** 1 Universitas Indonesia, Jakarta, Indonesia

Thank you for the opportunity to review the paper titled 'Supervision preparedness among GP registrars: A survey study'. The topic on clinical supervision and challenges being encountered by GP registrars is both important and interesting. 

 The paper holds potentials, yet also has rooms for substantial improvements. 

 1. The introduction has elaborated the principles of clinical supervisions including those in the interprofessional context. The introduction warrants strengthening in terms of GP training context in different countries and their roles in clinical supervision. Also, since the study involves GP registrars, description on the impact on the clinical supervision should be described more clearly. The gap analysis is rather unclear. For example, the focus on Wessex region: why is it important? 

 The research question should also be stated more clearly. 

 2. The methods of the study should include the study setting e.g the GP training setting in the UK and the Wessex region in more details. The development of the survey tool should be explained in more details, including the validation process of the instrument. The recruitment of the GP trainees/registrars should also be explained more clearly as well as the targeted sample size.

 I am not sure that the qualitative data analysis should be reconsidered, whether it is thematic analysis or content analysis. 

 3. The results section should be substantially constructed by elaborating the quantitative data: the characteristics of the respondents, the descriptive results of the quantitative data. The qualitative data should be supported by the quotes from the qualitative response.

 4. The discussion can be strengthened by further revision of the results as suggested.

Have any limitations of the research been acknowledged?

Partly

Is the work clearly and accurately presented and does it cite the current literature?

Partly

If applicable, is the statistical analysis and its interpretation appropriate?

Not applicable

Are all the source data underlying the results available to ensure full reproducibility?

Partly

Is the study design appropriate and is the work technically sound?

Partly

Are the conclusions drawn adequately supported by the results?

Partly

Are sufficient details of methods and analysis provided to allow replication by others?

Partly

Reviewer Expertise:

curriculum development, faculty development

I confirm that I have read this submission and believe that I have an appropriate level of expertise to state that I do not consider it to be of an acceptable scientific standard, for reasons outlined above.

# Response

**Published:** 2026-03-06

**Authors:** Ioannis Saxionis

**Affiliations:** Primary Care, University Hospital Southampton NHS Foundation Trust, Southampton, England, UK

We thank the reviewer for their careful reading of our manuscript and for the constructive feedback. We appreciate the recognition that the topic of clinical supervision among GP registrars is important and relevant. We have revised the manuscript to address the reviewer’s comments and to improve the clarity, context, and reporting of the study. Our responses are provided below:

**Comment 1:** The introduction should be strengthened with more context regarding GP training in different countries, the role of GP registrars in clinical supervision, clarification of the research gap, and the relevance of the Wessex region. The research question should also be clearer.

**Response:**  We agree that additional context would improve the framing of the study. The Introduction has been expanded to provide clearer background on clinical supervision within modern primary care systems and the increasing expectation that GP registrars supervise members of the multidisciplinary workforce. We have also added further explanation regarding the types of Allied Health Professionals commonly supervised in UK primary care (e.g., paramedics, nurses, advanced practitioners, and physician associates). The rationale for the study and the research gap have been clarified, highlighting that the study focus is on UK registrars and emphasising that while supervision responsibilities are increasingly expected in practice, formal preparation for this role during GP training remains variable.  We have also clarified the relevance of the Wessex region, noting that the higher response rate from this region reflects the authors’ training networks and dissemination pathways rather than a study design choice, and this is now acknowledged as a limitation.

**Comment 2: **The methods should describe the GP training setting in the UK and Wessex region in more detail. The survey development and validation process should also be described, and recruitment and sample size clarified. The qualitative analysis approach should also be clarified.

**Response:** The Methods section has been substantially expanded. We now provide additional details describing the UK GP specialty training programme, including the role of GP registrars and the clinical training environment. The development of the survey instrument is now described in greater detail, including the constructs measured (preparedness, confidence, barriers, and supervisory behaviours), the response scales used, and the process through which the survey was developed following a literature review. The questionnaire was also reviewed by senior academic GPs with expertise in medical education to assess face validity and content relevance prior to dissemination. As this was an exploratory survey, formal psychometric validation was not undertaken and this has now been explicitly acknowledged as a limitation. We have also clarified the recruitment process, explaining that the survey was disseminated through GP training networks and professional communication channels. Because multiple overlapping distribution routes were used, a precise denominator and formal response rate could not be calculated; this is now stated explicitly in the Methods and Limitations sections. Regarding qualitative analysis, we have clarified that a content analysis approach was used, rather than thematic analysis, and the manuscript has been revised accordingly.

**Comment 3: **The results section should elaborate more on quantitative findings and the qualitative data should include quotes.

**Response:** The Results section has been revised to present the quantitative findings more clearly, including expanded descriptions of the survey responses and respondent characteristics. Additionally, representative quotations from free-text responses have been incorporated to illustrate key themes identified in the qualitative analysis, improving transparency and supporting interpretation of the findings.

**Comment 4:** The discussion could be strengthened following revision of the results. **Response**: The Discussion section has also been revised to better align with the above additions and the limitation section has been expanded. We also further emphasise the exploratory nature of the study.

 We thank the reviewer again for their thoughtful and constructive feedback, which has helped us improve the clarity, methodological transparency, and overall presentation of the manuscript.

# Reviewer report: Reviewer response for version 1

**DOI:** 10.21956/mep.22676.r44211

**Published:** 2025-12-23

**Authors:** Devi Prasad Mohapatra

**Affiliations:** 1 Jawaharlal Institute of Postgraduate Medical Education & Research (JIPMER), Puducherry, Puducherry, India

The authors have carried out an interesting online survey, a cross-sectional study conducting a qualitative and quantitative assessment of clinical supervision experience, confidence, and perceptions of training among registrars involved in general practice. 

 Clinical supervision is a cornerstone of professional development and quality assurance in healthcare, particularly for general practitioners (GPs) and other health professionals. It is defined as a formal process of professional support and learning that enables individuals to:

 1. develop knowledge, competence, and confidence; 2. monitor their practice

 3. ensure the safety and well-being of their patients

 Clinical supervision will generally involve a regular, protected time for a supervisee to discuss their clinical work and professional challenges with a designated, experienced supervisor.(1)

 Effective clinical supervision is crucial for translating theoretical knowledge into practical skills, fostering reflective practice, reducing professional isolation, hence, ultimately improving patient care and outcomes.

 The present study involves:

1. Descriptive analysis of the collected data from the respondents
2. Thematic analysis of the free-text responses

The survey instrument used here may require expert validation to establish its psychometric properties. 

 Although the sample size is small, the authors have identified a significant lack of formal training in clinical supervision among the respondents. 'Time constraint' was a recurring theme among athe respondents whereas awareness of the concept was present among them,

 The generalizability of the findings is limited by the current sample size. A larger cohort study is necessary to draw concrete recommendations for curriculum development regarding clinical supervision.

 Stakeholders need to include clinical supervision as a formal curricular program to develop a more ready workforce. 

 References:

 1. Kilminster, S., Cottrell, D., Grant, J., & Jolly, B. (2007). AMEE Guide No. 27: Effective educational and clinical supervision. *Medical Teacher*, *29*(1), 2–19. https://doi.org/10.1080/01421590701210907

Have any limitations of the research been acknowledged?

Yes

Is the work clearly and accurately presented and does it cite the current literature?

Yes

If applicable, is the statistical analysis and its interpretation appropriate?

Yes

Are all the source data underlying the results available to ensure full reproducibility?

Yes

Is the study design appropriate and is the work technically sound?

Yes

Are the conclusions drawn adequately supported by the results?

Yes

Are sufficient details of methods and analysis provided to allow replication by others?

Partly

Reviewer Expertise:

Curriculum Development, continuing professional development, digital tools in medical training, simulation

I confirm that I have read this submission and believe that I have an appropriate level of expertise to confirm that it is of an acceptable scientific standard.

## References

[1] : AMEE Guide No. 27: Effective educational and clinical supervision. *Medical Teacher* .2007;29(1) : 10.1080/01421590701210907 2-19 10.1080/01421590701210907 17538823

# Response

**Published:** 2026-03-06

**Authors:** Ioannis Saxionis

**Affiliations:** Primary Care, University Hospital Southampton NHS Foundation Trust, Southampton, England, UK

We would like to thank the reviewer for their positive and constructive evaluation of our manuscript and for highlighting the relevance of clinical supervision in supporting professional development, reflective practice, and patient safety in healthcare.  We appreciate the reviewer’s recognition of the importance of the study and their acknowledgement that the work is clearly presented and supported by relevant literature. In response to the broader comments regarding methodological clarity and survey validation, we have made revisions in the updated version of the manuscript to further strengthen transparency and reporting.  In particular, the Methods section has been expanded to provide additional detail about the development of the survey instrument, including the constructs assessed (preparedness, confidence, barriers, and supervisory behaviours) and the response scales used. We have also clarified that the survey was developed following a review of existing literature and that it was reviewed by senior academic GPs with expertise in medical education to assess face validity and content relevance prior to dissemination. We also now explicitly state that, as this was an exploratory educational survey, formal psychometric validation and pilot testing were not undertaken, which is acknowledged as a limitation.  Minor revisions have also been made across the manuscript to improve clarity and consistency of terminology (e.g., use of “GP registrars” throughout) and to provide additional context in the Introduction regarding the multidisciplinary workforce in primary care.  We thank the reviewer again for their supportive assessment and helpful comments, which helped improve the clarity and transparency of the manuscript.
